# Spatial variation in risk for tick-borne diseases in residential areas of Dutchess County, New York

**DOI:** 10.1371/journal.pone.0293820

**Published:** 2023-11-09

**Authors:** Felicia Keesing, Emma Tilley, Stacy Mowry, Sahar Adish, William Bremer, Shannon Duerr, Andrew S. Evans, Ilya R. Fischhoff, Fiona Keating, Jennifer Pendleton, Ashley Pfister, Marissa Teator, Richard S. Ostfeld

**Affiliations:** 1 Bard College, Annandale, NY, United States of America; 2 Cary Institute of Ecosystem Studies, Millbrook, NY, United States of America; 3 Department of Behavioral and Community Health, Dutchess County, NY, United States of America; Cairo University Faculty of Veterinary Medicine, EGYPT

## Abstract

Although human exposure to the ticks that transmit Lyme-disease bacteria is widely considered to occur around people’s homes, most studies of variation in tick abundance and infection are undertaken outside residential areas. Consequently, the patterns of variation in risk of human exposure to tick-borne infections in these human-dominated landscapes are poorly understood. Here, we report the results of four years of sampling for tick abundance, tick infection, tick encounters, and tick-borne disease reports on residential properties nested within six neighborhoods in Dutchess County, New York, USA, an area of high incidence for Lyme and other tick-borne diseases. All properties were within neighborhoods that had been randomly assigned as placebo controls in The Tick Project; hence, none were treated to reduce tick abundance during the period of investigation, providing a unique dataset of natural variation within and between neighborhoods. We estimated the abundance of host-seeking blacklegged ticks (*Ixodes scapularis*) in three types of habitats on residential properties–forests, lawns, and gardens. In forest and lawn habitats, some neighborhoods had consistently higher tick abundance. Properties within neighborhoods also varied consistently between years, suggesting hot spots and cold spots occurring at a small (~ 1-hectare) spatial scale. Across neighborhoods, the abundance of nymphal ticks was explained by neither the amount of forest in that neighborhood, nor by the degree of forest fragmentation. The proportion of ticks infected with three common tick-borne pathogens did not differ significantly between neighborhoods. We observed no effect of tick abundance on human encounters with ticks, nor on either human or pet cases of tick-borne diseases. However, the number of encounters between ticks and outdoor pets in a neighborhood was negatively correlated with the abundance of questing ticks in that neighborhood. Our results reinforce the need to understand how human behavior and neglected ecological factors affect variation in human encounters with ticks and cases of tick-borne disease in residential settings.

## Introduction

The abundance of ticks and their prevalence of infection with tick-borne pathogens are expected to be key determinants of the risk for human exposure to tick-borne diseases, including Lyme disease [1, 2]. Many studies have documented dramatic variation between locations and through time in the abundance of infected *Ixodes* ticks, and consequently in risk of human exposure to the pathogens they transmit, including the agents of Lyme disease, human babesiosis, and human granulocytic anaplasmosis (e.g., [3–5]). The relevance of this variation in tick abundance and infection prevalence to patterns of tick-borne disease in humans, however, is not straightforward. Sampling of tick abundance and infection prevalence often occurs in habitats or landscapes that are relatively free of direct human disturbance, such as native forests in rural or exurban landscapes [6–8]. However, considerable evidence suggests that human exposure to Lyme disease frequently occurs in residential areas [9–11]. Hence, study sites selected for estimating tick-borne disease risk are often distinct from the places where people live and engage in outdoor activities. As a result, estimates of variation in abundance of infected ticks in space and time in non-residential areas may not correlate with actual human exposure.

A recent meta-analysis [12] revealed that ecological and behavioral factors associated with increased probability of tick-borne disease were detectable at the scales of the residential property and the neighborhood (defined as the zone within 500 meters of the property), and also at a broader scale beyond the neighborhood. Studies at the scale of the residential neighborhood were less well-represented in the meta-analytic database than were studies at the scale of the individual yard or outside the neighborhood [12]. Most of the risk factors in the studies analyzed by Fischhoff et al. [12] were human behaviors, such as the use of tick repellents and protective clothing, or the conduct of tick checks, as well as participation in outdoor activities (e.g., picnicking, yard work). Some ecological variables, such as the presence of woodpiles, rock walls, and nearby forests, were also examined. Most studies were also short-term (≤1 year) [12]. Thus, prior analyses have frequently not explored spatial and temporal patterns of variation in abundance and infection prevalence of *Ixodes* ticks in the locations where human exposure is likely high, nor have they addressed ecological correlates of those patterns at that scale [13, 14].

Our goal in this study was to increase understanding of the patterns of variation in risk of exposure to ticks and tick-borne pathogens within residential neighborhoods. We focused on variation between individual properties within neighborhoods, variation between neighborhoods, and variation between years. Thus, we explored whether “hot spots” and “cold spots” could be detected and whether they persisted through time. We asked whether the variation in both tick abundance and infection prevalence with tick-borne pathogens was associated with variation in tick encounters and cases of tick-borne disease in both human and outdoor-pet residents of the neighborhoods.

We predicted that some neighborhoods would have consistently more host-seeking nymphal ticks than others did. We also predicted that some individual properties within neighborhoods would have consistently more host-seeking nymphs. We expected that neighborhoods with more forest would maintain higher tick populations, and also that individual properties with more forest would maintain higher tick populations. We also hypothesized that neighborhoods would vary in the average proportion of ticks infected with tick-borne pathogens. Finally, we predicted that the abundance of ticks in neighborhoods would correlate positively with tick encounters and self-reported cases of tick-borne diseases for both people and outdoor pets.

## Methods

We collected data on tick abundance and infection, human and pet encounters with ticks, and human and pet cases of tick-borne diseases, for six residential neighborhoods in Dutchess County, New York, USA from 2017–2021. These neighborhoods were part of The Tick Project, a randomized, placebo-controlled, double-masked experiment to test the effects of two methods of tick control–Met52 fungal spray (Novozymes) and MaxForce TCS bait boxes [15]. The six neighborhoods analyzed here comprised the controls for this experiment. Enrolled properties in these neighborhoods were treated with placebo interventions, i.e. water instead of fungal spray, and bait boxes containing no acaricide. Thus, they provide baseline data on unmanipulated tick abundance and infection, as well as tick encounters and tick-borne disease incidence, in untreated residential neighborhoods.

Each neighborhood consisted of approximately 100 properties, averaging ~0.2 hectares each. Details of participant recruitment are provided in Keesing et al. [15]. We excluded properties where residents were not willing to forgo the use of acaricides and insecticides for the duration of the study.

We characterized the habitats of each neighborhood overall (e.g. proportion of forest), and of each property participating in the study. To estimate the habitat characteristics of the neighborhoods, we used publicly-available digital orthoimagery of Dutchess County (New York State Digital Orthoimagery Program 2016) taken in the spring of 2016. We classified every pixel in each neighborhood as either habitat (forest, field, lawn, shrub/garden) or impervious surface (e.g. house, shed, driveway, road) using the maximum likelihood classification tool in ArcMap software (version 10.4). Further details are described in Keesing et al. [15]. We calculated the degree of forest fragmentation in our neighborhoods using FragStats version 4.2 [16]. Edges of forest patches were truncated at neighborhood boundaries, and we used FragStats to calculate the mean patch area for each neighborhood, and the fractal dimension index, which characterizes how patch perimeter increases per unit increase in patch area.

### Tick abundance

We collected data on tick abundance and infection from 20 properties in each neighborhood during the peak period of nymphal activity (May-June [5]) for four years (2017–2019, 2021), sampling each property twice each year. The specific properties we sampled varied somewhat from year to year, based on permissions from property owners. Here, for estimates of tick abundance, we include only those properties that we were able to sample every year–an average of 13.7 (range 12–16) in each neighborhood.

To estimate the abundance of questing nymphal ticks, we conducted up to ten 30-second flagging intervals in each habitat type (forest, lawn, shrub/garden), using a 1 m x 1 m white corduroy cloth, inspecting the cloth for ticks after each interval [15]. The number of intervals was proportional to the area of the habitat type on an individual property, with a maximum of ten intervals for each habitat type. Researchers included the ticks observed on their own field attire at the end of each flagging interval. Tick sampling was conducted between 0900h and 1730h, and we did not sample when vegetation was wet.

### Tick infection

Nymphal ticks collected during surveys of tick abundance were stored alive in humidified vials, with all ticks from a neighborhood pooled each year, regardless of the property and habitat type from which the tick was collected. Within three weeks of collection, we surface-sterilized the ticks with 10% bleach (sodium hypochlorite), rinsed them with deionized water, and then stored them individually at -80°C. We used real-time PCR to detect the presence of three pathogens–*Borrelia burgdorferi* (causative agent of Lyme disease), *Anaplasma phagocytophilum* (anaplasmosis), and *Babesia microti* (babesiosis), following procedures described in detail in Ostfeld et al. [17].

### Case and encounter data

Recruitment of participants occurred between 6 May, 2016 and 31 May, 2017. Participants agreed to provide informed consent in writing. From 2017–2020, we distributed biweekly surveys to the primary contact person for each participating household, as described in detail in Keesing et al. [15], asking whether anyone in the household, including pets that spent time outdoors, had encountered a tick or been diagnosed with a tick-borne illness in the preceding two weeks. When participants answered yes to that question, we prompted them to provide further details. The institutional review board of the Cary Institute of Ecosystem Studies in Millbrook, NY, USA reviewed and approved all protocols involving human subjects (#131–2016).

### Data analysis: Tick abundance

We analyzed all data with R (version 4.0.1), using the packages *dplyr*, *forcats*, *ggplot2*, and *cowplot* [18–22] for data manipulation and presentation. For all statistical models, we used package *DHARMa* to compare the fit of our data to the assumptions of the models [23].

To test the hypothesis that neighborhoods varied in the abundance of questing nymphal ticks (H1) in forested areas of properties, we analyzed the maximum number of nymphal ticks per flagging interval, including only the larger of the two season-specific estimates of nymphal abundance on each property. We constructed a linear model, with neighborhood and the proportion of forest cover on the property as predictors. For lawns and gardens, our data on the abundance of nymphal ticks per flagging interval were zero-inflated, with a substantial proportion (64–71%) of our sampling in these two habitats yielding no ticks. As a result, to test H1 for these habitat types, we used a hurdle modeling approach, first fitting a model to the sites with and without ticks, and then attempting to fit a model to the abundance of ticks at sites where we they did occur in our samples. For these habitats, we used generalized linear mixed models (glmm) with a binomial distribution and a logit link function to fit a logistic model to the zero versus non-zero data. We included neighborhood and year as fixed effects and property nested within neighborhood as a random effect. Here, replicates were the properties sampled each year (2017, 2018, 2019, and 2021). We used the *glmmTMB* function in package *glmmTMB* [24] to conduct these analyses. We analyzed the fit of our models with the *Anova* function in package *car* [25]. Because the non-zero values did not conform to the assumptions of statistical distributions (e.g. gamma, gaussian), we did not fit models to these data. We did not include the proportion of each habitat type (e.g. proportion of lawn) on a property in our glmm models because our sampling intensity (number of flagging intervals) was proportional to the size of each habitat on a property (e.g. more intervals in a larger lawn). As a result, our probability of detecting ticks (our response variable in these analyses) was potentially confounded by the proportion of a property comprising each habitat type.

To test the hypothesis that individual properties differed in the presence of questing nymphal ticks (H2), we used a generalized linear mixed model with the presence of ticks in forested habitat on an individual property as the dependent variable, considering the four years of the study as replicates. We included the individual property as a fixed effect, and the neighborhood in which the property occurred as a random effect, using a binomial distribution. We compared the fit of this model to a null model without the inclusion of individual properties using analysis of variance.

To test the hypothesis that neighborhoods with higher percent cover of forest would maintain higher tick populations (H3), we built a linear regression model for each habitat type with the proportion of the neighborhood that was forest as the independent variable and the mean abundance of questing nymphal ticks in the habitat as the dependent variable.

To test the hypothesis that properties with higher percent cover of forest would maintain higher tick populations (H4), we assessed whether the proportion of forest on a property affected the average abundance of nymphal ticks collected across years on a property, for each habitat type separately. For this, we used linear models, with the mean number of nymphs per interval in a habitat type as the dependent variable, and neighborhood and the proportion of forest on the property as factors. Data were transformed to conform to assumptions of tests.

At the individual property level, we used a chi-square test to evaluate whether there was an association between the detection of ticks in forest and the detection of ticks on lawns of the same properties.

### Data analysis: Tick infection

To test the hypothesis that tick infection varied between neighborhoods (H5), we used linear models to analyze the proportion of ticks in a neighborhood infected with each of the three tick-borne pathogens. Our models included neighborhood as a fixed effect, and used years (2017–2019, 2021) as replicates. The proportion of ticks infected with each pathogen was log-transformed to meet assumptions of tests.

### Data analysis: Tick encounters and cases

To test the hypothesis that the abundance of ticks in neighborhoods would correlate positively with tick encounters and self-reported cases of tick-borne diseases for both people and outdoor pets (H6), we used generalized linear models to determine whether encounters and cases of tick-borne diseases for humans and pets differed significantly among neighborhoods, using years (2017–2020) as replicates. In these models, we used either a poisson or a negative binomial distribution, with neighborhood as a fixed effect, and with an offset for the number of people (or pets) in the neighborhood each year.

To assess whether tick encounters or disease cases were correlated with the annual abundance of ticks in forested habitats, we used linear mixed-effect models with the mean number of nymphal ticks per interval as a fixed effect and neighborhood as a random effect.

## Results

### Tick abundance

**Fig 1:**
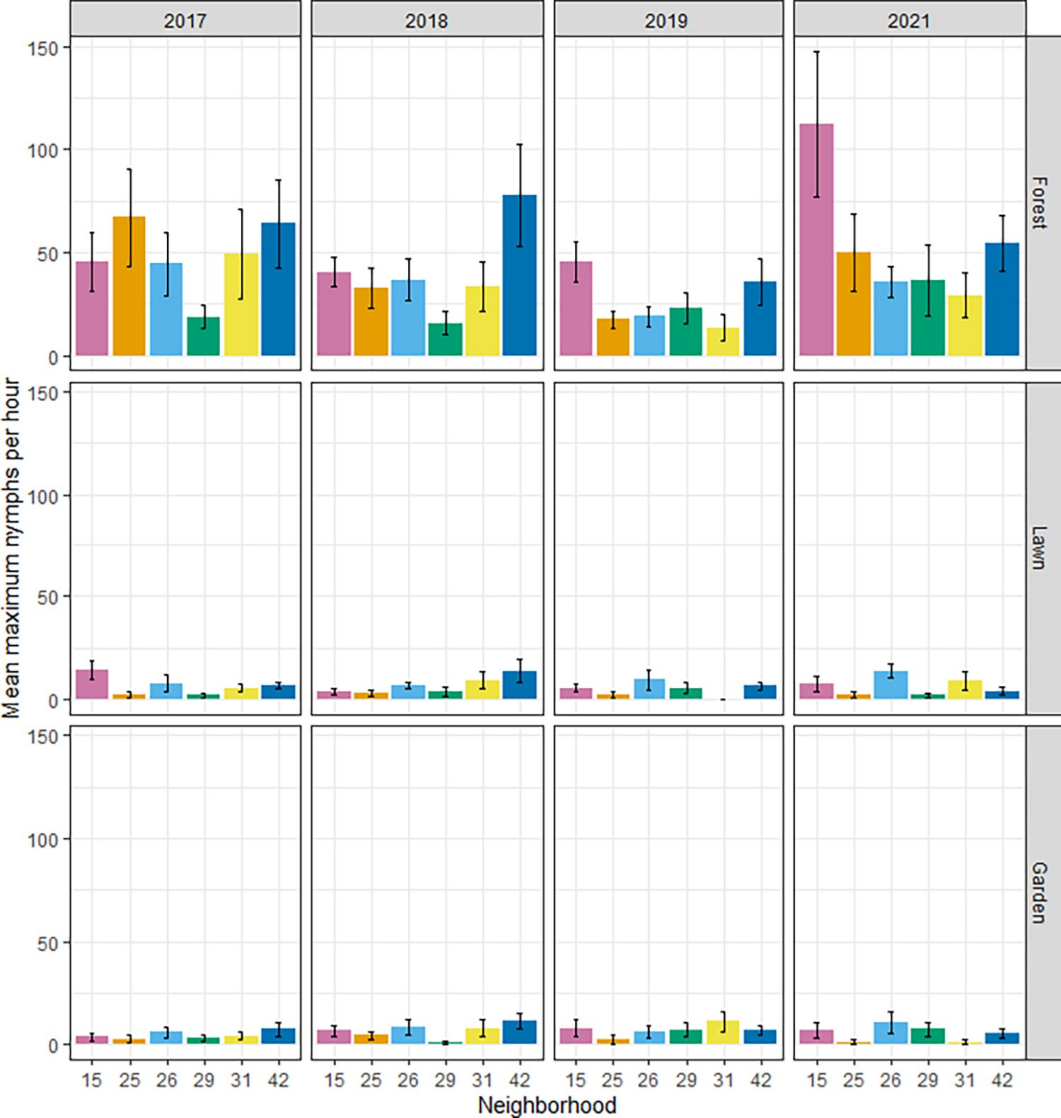
Mean number of nymphal ticks per hour in each of six residential neighborhoods of Dutchess County, New York, USA. Values represent averages of sampled properties in each neighborhood for each of three habitat types (rows) in each of four years (columns); error bars represent standard errors. Properties were not sampled for ticks in 2020 because of the COVID-19 pandemic.

The data described here were collected on 71 residential properties distributed in six neighborhoods in Dutchess County, New York, with each property sampled for four years (2017, 2018, 2019, and 2021). Approximately 99% of ticks were identified as *Ixodes scapularis* [15]. Forested areas of properties had more ticks than either lawns or gardens did (Fig 1, S1 Fig). Supporting hypothesis H1, the abundance of ticks in forests varied significantlyamong neighborhoods (F_5,63_ = 2.74, P = 0.03), with neighborhood 29, for example, having approximately one-third the number of ticks on average that neighborhood 15 did (Fig 1). The presence of ticks in lawns also varied significantly among neighborhoods (χ^2^_5_ = 13.3, P = 0.02), with neighborhood 29 having the fewest properties with ticks detected in their lawns (Fig 1, S1 Fig). We did not detect significant differences among neighborhoods in the presence of ticks in gardens (χ^2^_5_ = 6.7, P = 0.25), and there were no significant effects of year on tick abundance in any of the habitat types.

Supporting hypothesis H2, we found that some individual properties consistently had ticks, while others consistently did not (S4 Fig). Using years as replicates, we observed significant differences between individual properties that affected the presence of ticks in forested habitats, when neighborhood was taken into account (χ^2^_70_ = 149.26, P<<0.01).

**Fig 2:**
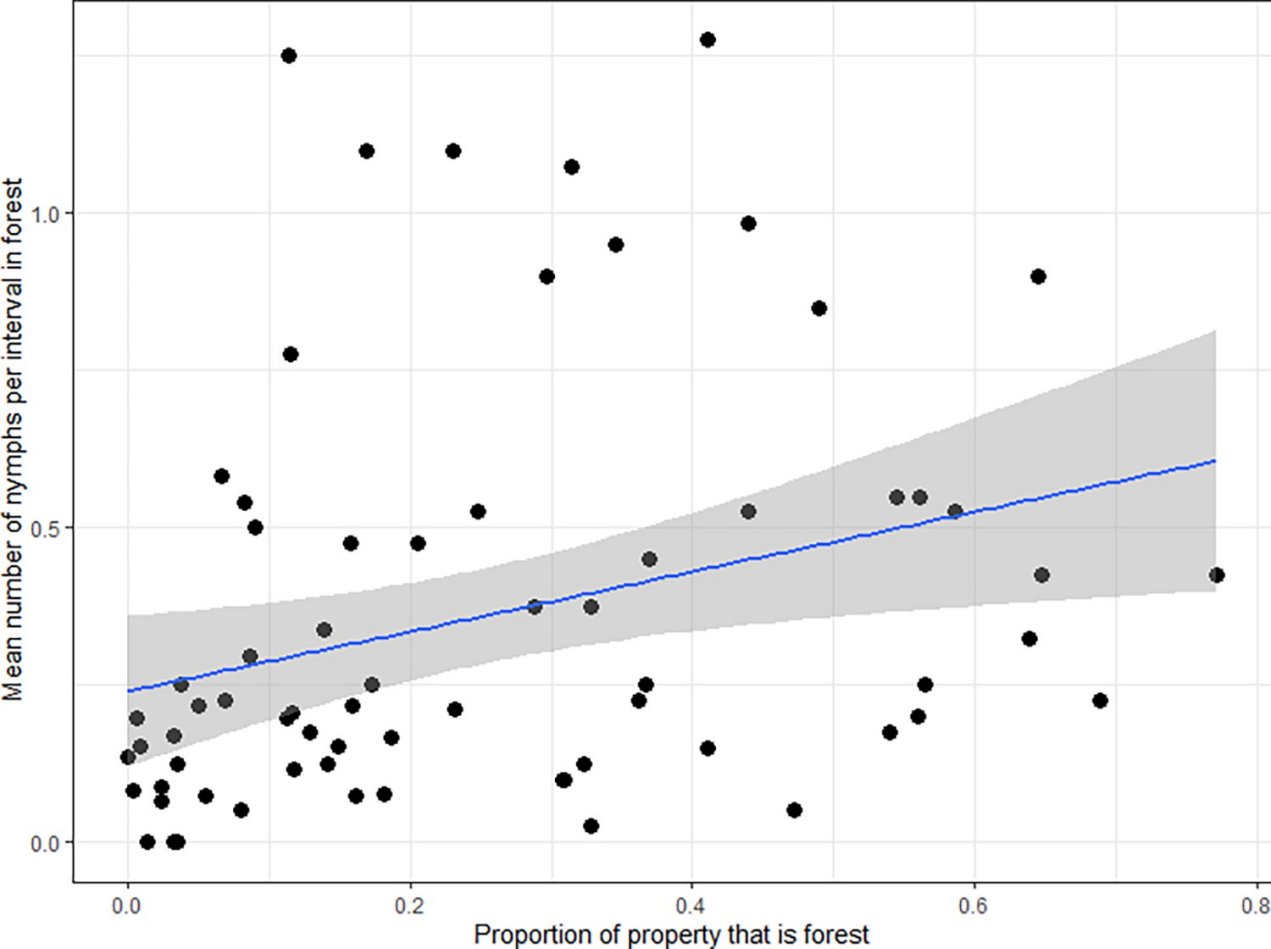
Mean number of nymphal ticks per 30-second flagging interval in properties in each of six residential neighborhoods of Dutchess County, New York, USA, versus the proportion of each property that was forested. Y-values represent the number of ticks in forested areas of each property averaged over the four years of sampling (2017–2019, 2021). Line represents the line of best fit, and shading represents 95% confidence intervals.

Supporting hypothesis H4, we found that the abundance of ticks per flagging interval in forested areas of properties was positively correlated with the proportion of each property that was forested (F_1,63_ = 11.74, P = 0.001, Fig 2). However, this proportion explained relatively little of the variation in abundance of ticks (R^2^ = 0.2), with 80% of the variation explained by other unknown factors. On average, 45% (range 36–63%) of the entire neighborhood was forested (S1 Fig). Contrary to hypothesis H3, we found that this forest coverage at the neighborhood level was not significantly correlated with the average abundance of ticks in the forests (P = 0.73), lawns (P = 0.34), or gardens (P = 0.33) of sampled properties within the neighborhoods. Thus, the correlation between forest cover and tick abundance observed when comparing individual properties was not observed when comparing neighborhoods.

Neighborhoods contained an average of 32 forest patches (range 14–60), with a mean patch size of 0.54 ha (range 0.29–1.5 ha). The mean abundance of ticks in forested habitats was not significantly correlated with the degree of fragmentation of forest in the neighborhoods (F_1,4_ = 0.51, P = 0.51).

Overall, ticks were detected in forests of 77% of properties, but only in 38% of lawns (Table 1). The presence of ticks in forested areas of properties was significantly associated with the detection of ticks on lawns of those same properties, such that there was a higher probability of detecting ticks in either both habitat types, or neither, than expected by chance (χ^2^_1_ = 6.51, P = 0.01; Table 1).

**Table 1:** Observed frequencies of the presence of ticks on individual properties in forest and lawn habitat types. Properties were significantly more likely to have ticks detected in both forest and lawn, or to have no ticks detected in either. Values in parentheses are expected values assuming no association between habitat types.

	No ticks in lawn	Ticks in lawn
**No ticks in forest**	17% *(14%)*	6% *(9%)*
**Ticks in forest**	44% *(46%)*	33% *(29%)*

### Tick infection

**Fig 3:**
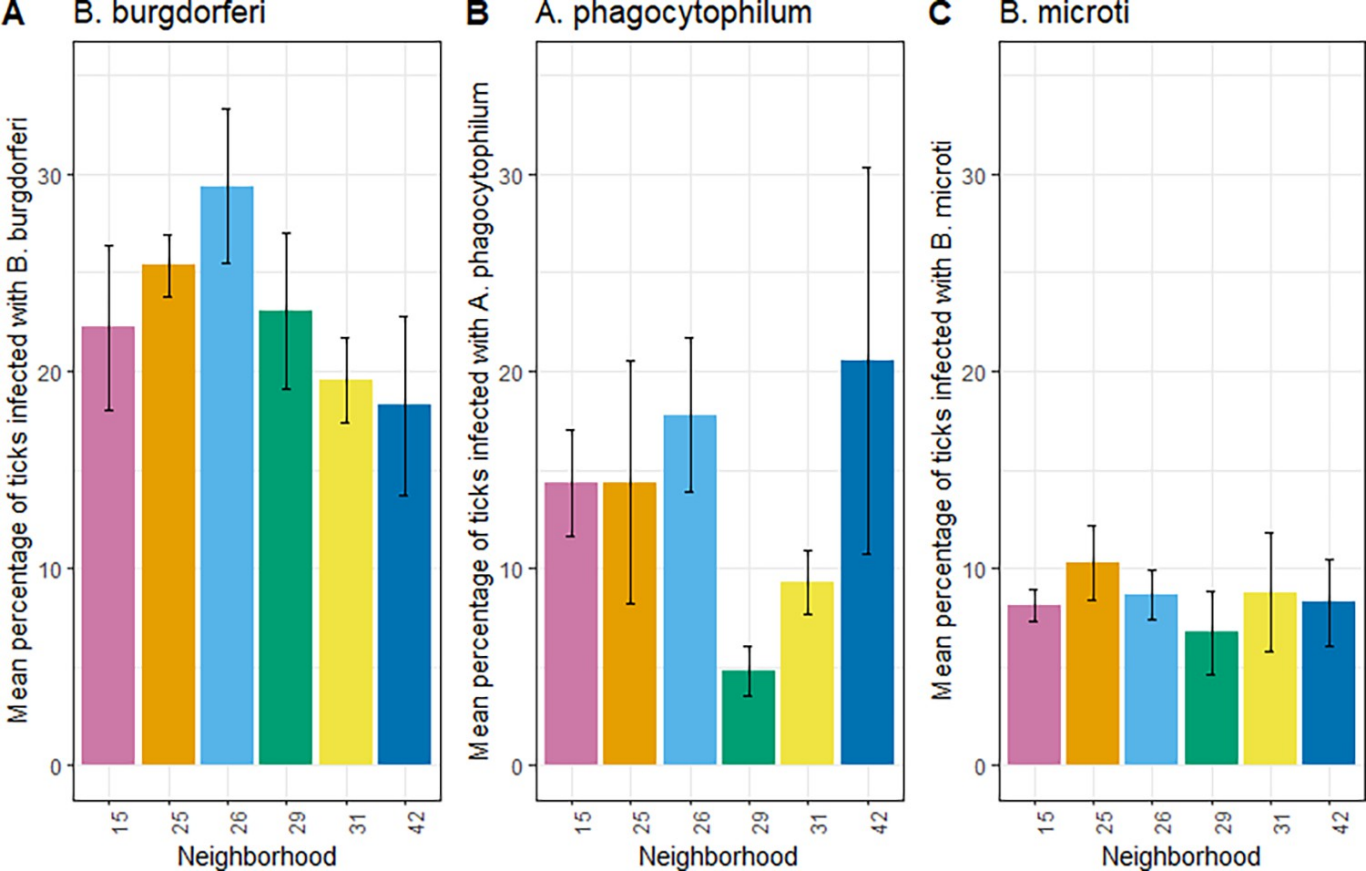
Mean percentage of ticks infected with (A) *Borrelia burgdorferi*, (B) *Anaplasma phagocytophilum*, and (C) *Babesia microti* in six residential neighborhoods in Dutchess County, New York, USA. Y-values represent the mean infection from four years of sampling (2017–2019, 2021), and error bars represent standard error of the mean.

On average, 23% (range 18–29%) of ticks in the neighborhoods were infected with *B*. *burgdorferi*, while 14% (5–18%) were infected with *A*. *phagocytophilum*, and 8% (7–10%) with *B*. *microti* (Fig 3). Contrary to hypothesis H5, these proportions did not vary significantly among neighborhoods for any of the pathogens (*Bb*: F_1,22_ = 1.54, P = 0.23; *Ap*: F_1,22_ = 0.15, P = 0.70; *Bm*: F_1,22_ = 0.02, P = 0.88).

### Tick encounters and cases

**Fig 4:**
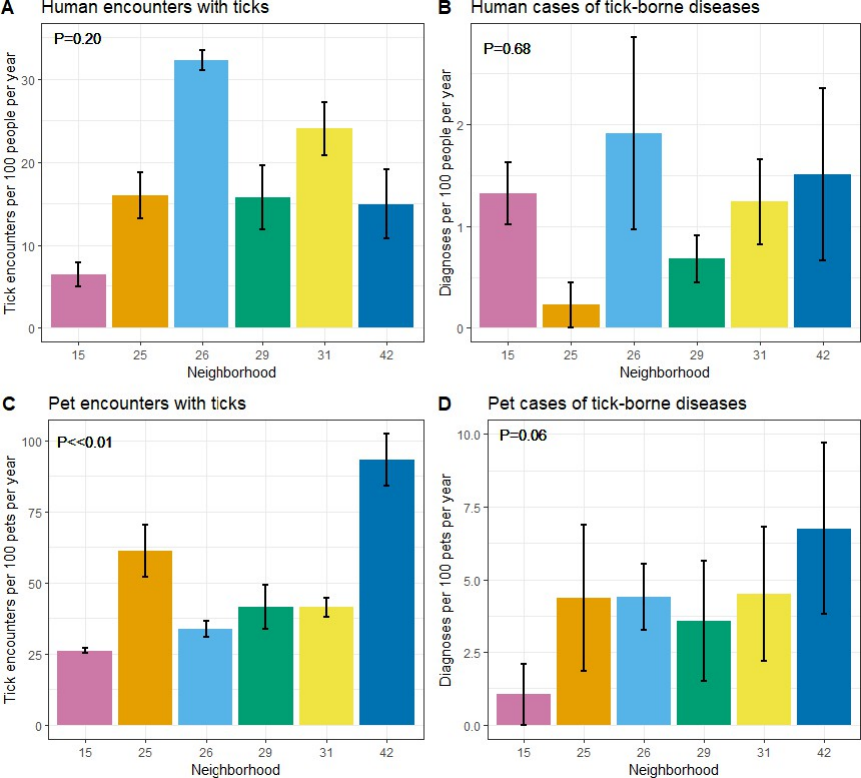
Mean (A) encounters of humans with ticks, (B) human cases of tick-borne diseases, (C) encounters of outdoor pets with ticks, and (D) pet cases of tick-borne diseases as reported by study participants on six residential neighborhoods in Dutchess County, New York, USA. Y-values represent the mean annual number of encounters/cases per 100 people/pets over four years of sampling (2017–2019, 2021). Error bars represent standard error of the mean.

People in the neighborhoods reported on average ~18 (range 6–32) tick encounters per year for every 100 people. Participants in neighborhood 15 reported the fewest encounters, which occurred at a rate five times lower than in neighborhood 26. Contrary to hypothesis H6, the per-capita encounter rate did not differ significantly overall between neighborhoods (χ^2^_1_ = 1.63, P = 0.20; Fig 4A). Neighborhoods did not differ significantly in incidence of diagnosed cases of tick-borne diseases (χ^2^ = 0.17, P = 0.68), as reported by participants, with an overall average of approximately one diagnosed case per year for every 100 people (Fig 4B).

For outdoor pets living in the six neighborhoods, ~50 tick encounters occurred (range 26–93) per 100 pets each year, based on reports from participants (Fig 4C). The probability of these pet encounters with ticks, as reported by participants, differed significantly between neighborhoods (χ^2^ = 27.70, P <<0.01). On average, participants reported 4 (range 1–7) cases of tick-borne diseases in their pets each year for every 100 pets, and this per capita incidence did not differ significantly among neighborhoods (χ^2^ = 3.5, P = 0.06; Fig 4D).

We detected no statistically significant correlations between the mean abundance of nymphal ticks in forests in a neighborhood in a given year and either reported tick encounters (χ^2^ = 1.60, P = 0.21) or reported cases (χ^2^ = 0.95, P = 0.33) for people. Unexpectedly, reported encounters of pets with ticks were negatively correlated with the average number of nymphal ticks in forests in a neighborhood (χ^2^ = 14.79, P<<0.01), such that there were more per-capita encounters when there were fewer ticks (S4 Fig). However, there was no significant effect of tick abundance in forest on reported pet cases of tick-borne disease (χ^2^ = 0.10, P = 0.75).

## Discussion

This study took place in residential neighborhoods in the Hudson Valley of New York, which has experienced high incidence of Lyme disease and other tick-borne diseases for several decades [26]. By sampling abundance of nymphal *Ixodes scapularis* ticks, and their infection prevalence with three zoonotic pathogens, on 71 individual properties in six neighborhoods over four years, we were able to estimate variation in risk of human and pet exposure to tick-borne pathogens through space and time. We further pursued whether specific habitat variables explained the observed risk, and whether variation in risk was associated with tick encounters and cases of tick-borne disease in people and their outdoor pets. This work took advantage of the six control neighborhoods of The Tick Project [15, 27]. Unlike the other 18 neighborhoods of The Tick Project, these six neighborhoods received no acaricidal treatments; thus tick abundance and infection prevalence were monitored but not manipulated.

We found support for our prediction that some neighborhoods would have consistently more host-seeking nymphal *I*. *scapularis* ticks. In forest and lawn habitats within these neighborhoods, variation between neighborhoods was statistically significant, whereas variation in gardens was not, and these patterns were maintained across the four years of the study. Therefore, at the scale of residential neighborhoods, we detected the occurrence of hot spots and cold spots, defined respectively as neighborhoods with consistently higher than average, or lower than average, risk of human exposure to ticks. We also observed consistent differences in the abundance of questing ticks between individual properties, after statistically controlling for variation at the scale of neighborhoods, a finding that supports our second prediction. Our observation that the presence of ticks on lawns correlated positively with the presence of ticks in forest on the same property further supports the predictability of tick hot spots at the property scale. We did not detect significant differences between neighborhoods in nymphal infection prevalence with *B*. *burgdorferi*, *A*. *phagocytophilum*, or *B*. *microti*. This suggests that variation in exposure risk between neighborhoods is largely the result of variation in tick abundance, but not tick infection.

Given the occurrence of consistently higher- and lower-risk neighborhoods, and of properties within neighborhoods, we pursued both potential causes and consequences of those patterns. Many prior studies of blacklegged ticks in eastern North America have found higher abundances in forest than in other habitat types (e.g., [3, 28, 29]). Higher tick abundances in forests than in lawns or gardens were also detected in the current study and in prior [15, 17, 30] analyses of data from The Tick Project [27]. The presence of forest has frequently been postulated as a risk factor for Lyme disease and other tick-borne diseases [3, 11–13]. Here, we found no support for our prediction that forest cover within a neighborhood would be associated with the abundance of nymphal ticks within that neighborhood. Specifically, the abundance of host-seeking nymphs in forest, lawn, and garden within a neighborhood did not correlate with total forest cover within that neighborhood. We urge caution in interpreting this result, however, because of the truncation of land cover data at neighborhood boundaries. Comparing individual properties within neighborhoods, we detected a significant correlation between tick abundance in forest habitat and the proportion of the property consisting of forest. Despite this significant correlation, forest cover on the property explained only 20% of the variation between properties in tick abundance. Fragmentation statistics describing the spatial distribution of forest were not correlated with tick abundance within or between neighborhoods. These results suggest that vigorous pursuit of other ecological factors responsible for variation in tick abundance at the property level is warranted.

The epidemiological relevance of variation in abundance of host-seeking *I*. *scapularis* nymphs is somewhat controversial [10, 31]. Theoretical and correlational studies generally postulate positive associations [32–35]. For example, Mather et al. [32] detected a strong, positive correlation between abundance of infected blacklegged ticks and Lyme disease incidence between residential communities; Stafford et al. [33] reported similar positive correlations between years in specific locations in the northeastern United States; and Pepin et al. [36] reported strong associations between acarologic risk and Lyme disease incidence in across 36 eastern states. Although a correlation between tick abundance and cases of tick-borne disease is expected, several factors can weaken this relationship [10]. First, the shape of the relationship is poorly understood. If the relationship between tick abundance and cases of tick-borne disease is not linear, and particularly if there are sharp thresholds, correlations could be difficult to detect [31]. Second, human behaviors might disrupt a direct relationship between tick abundance and cases of disease. For example, if high prior incidence of tick-borne diseases in an area increases avoidance and self-protective behaviors, the expected correlation could be altered. Third, dissociations between the locations where tick abundance is estimated and where epidemiological outcomes are measured could undermine the ability to detect meaningful correlations. Any of these possibilities could help explain why experimental reductions in abundance of ticks using acaricides have not detected effects on encounters with ticks or cases of tick-borne disease in people [10, 15, 37].

In this study, despite a 5-fold difference between neighborhoods with the highest and lowest numbers of human encounters with ticks per-capita, we observed no significant relationship between tick abundance within a neighborhood and either encounters with people or reported cases of tick-borne diseases of residents therein. These results reinforce the conclusions from Keesing et al. [15] and Ostfeld et al. [30], which incorporated the 18 neighborhoods that received one or two tick-control interventions during The Tick Project. The lack of correlation between tick abundance and either encounters or cases in human residents was thus observed both in comparisons of the experimentally treated neighborhoods [15, 30] and in the current assessment of control (untreated) neighborhoods only.

For outdoor pets, we observed no significant correlation between tick abundance and reported cases of tick-borne disease in outdoor pets. Further, our observation of a significantly *negative* correlation between tick abundance and reported encounters of ticks on outdoor pets is directly contrary to our prediction. Unmeasured human behaviors toward pets could be responsible for this latter, unexpected association. For example, if pet owners increase their efforts to protect their pets (e.g., with acaricidal treatments or altering access to the yard) when perceived risk is highest, the expected positive relationship between tick abundance and tick encounters could be undermined or even reversed. More generally, the contrasts between pet and human responses to variation in tick abundance shown here and in Keesing et al. [15] suggest that differences in habitat use and behavior between these two groups of hosts are worth further study.

Our results reinforce the need to pursue human behavioral and ecological factors that affect variation in human encounters with ticks and cases of tick-borne disease in residential settings. Variable tick abundance is widely expected to affect risk and incidence, but the lack of support arising from both comparative and experimental approaches presents opportunities to pursue behavioral and neglected ecological factors in future efforts.

## Supporting information

S1 Data Data on control (untreated) neighborhoods in The Tick Project for 2017–2021 for six neighborhoods. Forest, Lawn, and Garden represent the mean number of nymphal ticks collected in each of the respective habitats, averaged across all of the sampled properties in a neighborhood, as described in the Methods. No tick data were collected in 2020. The total number of human and pet encounters and diagnoses represents the total for the entire neighborhood for a given year, with no data on these metrics collected in 2021 (details provided in Methods). Data on the number of humans and pets living in the neighborhood in each year, and the number of households enrolled in the study are provided, as is the average proportion of households responding to our biweekly surveys. The per capita rate of human and pet encounters, and diagnoses, is calculated for each year. For each neighborhood, we include the proportion of forested area in the neighborhood, as described in detail in the Methods.
(CSV)

S2 Data (CSV)

S1 Fig Habitat characteristics of the six neighborhoods based on percent forest, lawn/garden, and impervious surfaces. (TIF)

S2 Fig Mean number of nymphal ticks per hour in each of six residential neighborhoods of Dutchess County, New York, USA. Values represent averages of sampled properties in each neighborhood for each of three habitat types (rows) in each of four years (columns); error bars represent standard errors. Properties were not sampled for ticks in 2020 because of the COVID-19 pandemic. These are the same data as in Fig 1, but with a variable y-axis.
(TIF)

S3 Fig Mean number of nymphal ticks per 30-second flagging interval in forest habitats of the properties (x-axis) in each of six residential neighborhoods (columns) of Dutchess County, New York, USA over four years (rows). Values represent the mean maximum number of nymphal ticks per flagging interval in forested habitats. Properties were not sampled for ticks in 2020 because of the COVID-19 pandemic.
(TIF)

S4 Fig Reported tick encounters per 100 outdoor pets for each of six residential neighborhoods in Dutchess County, New York, versus the mean maximum number of nymphal ticks per flagging interval in forested habitats of properties in those neighborhoods. Colors represent the six neighborhoods sampled in three years (2017–2019). Tick encounters were negatively correlated with average tick abundance (χ^2^ = 14.79, P<<0.01).
(TIF)
