# Supplementary figures and images for: Spatial variation in risk for tick-borne diseases in residential areas of Dutchess County, New York
Source: PLoS One. 2023 Nov 9;18(11):e0293820. doi: 10.1371/journal.pone.0293820 (PMC10635528; doi:10.1371/journal.pone.0293820)

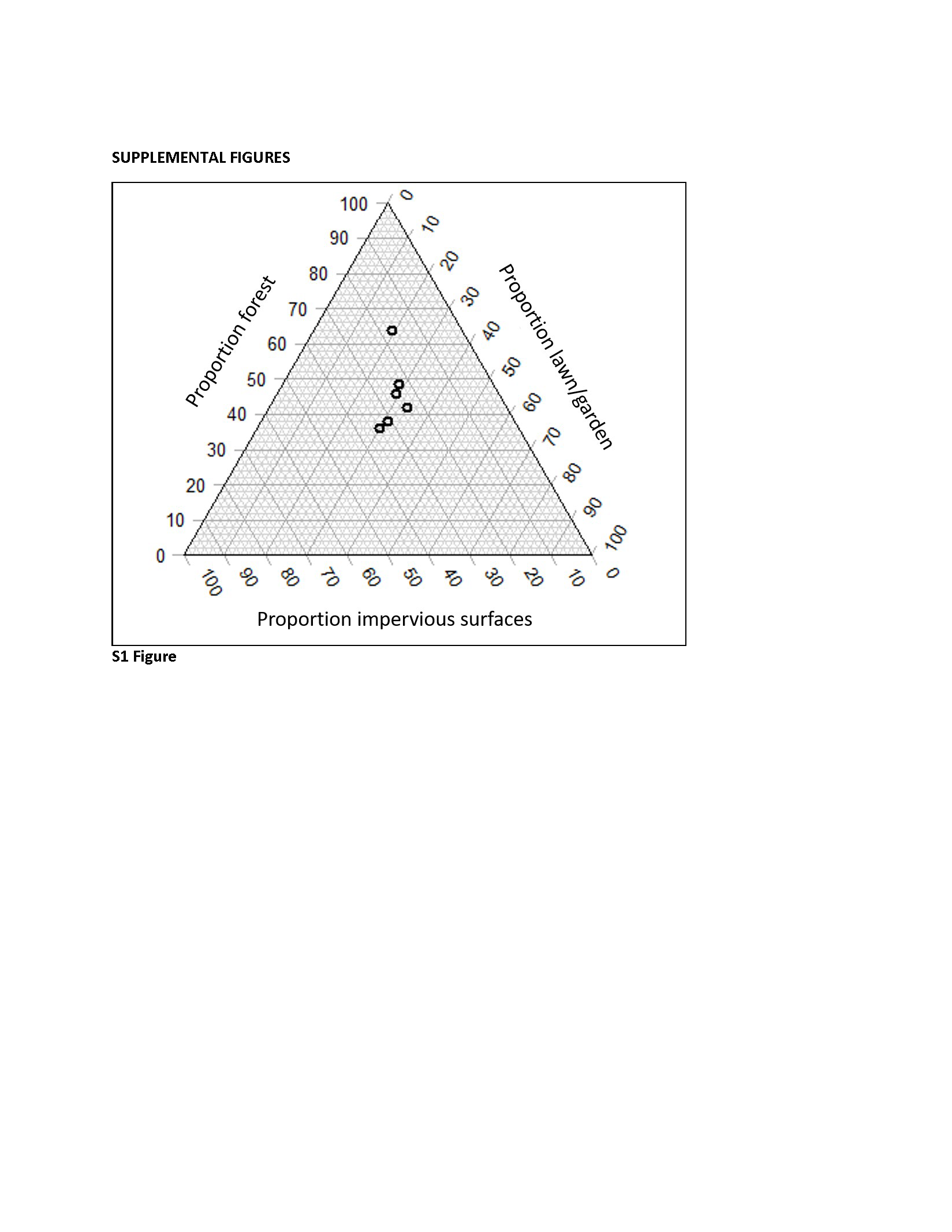

Supplement: S1 Fig — (TIF) [file pone.0293820.s003.tif]

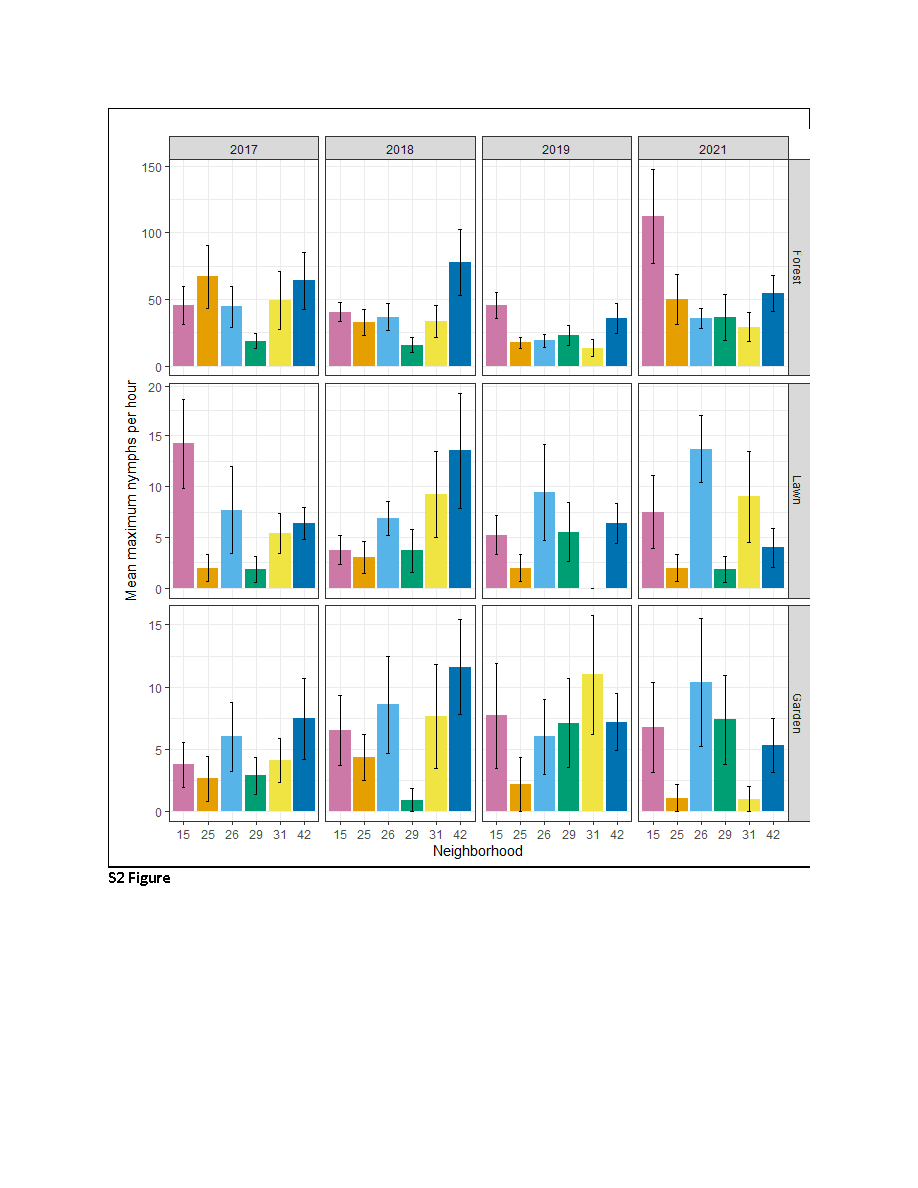

Supplement: S2 Fig — Values represent averages of sampled properties in each neighborhood for each of three habitat types (rows) in each of four years (columns); error bars represent standard errors. Properties were not sampled for ticks in 2020 because of the COVID-19 pandemic. These are the same data as in Fig 1, but with a variable y-axis. (TIF) [file pone.0293820.s004.tif]

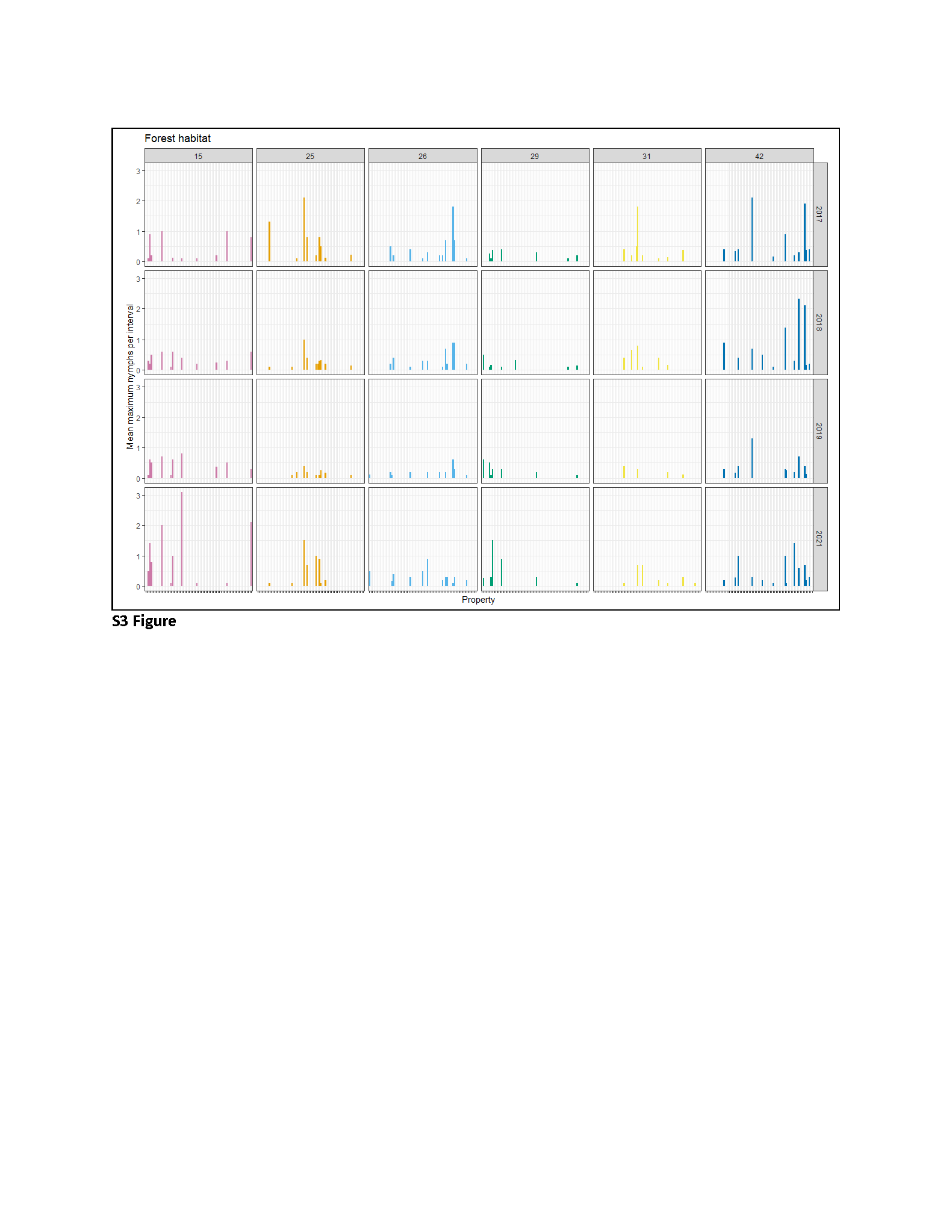

Supplement: S3 Fig — Values represent the mean maximum number of nymphal ticks per flagging interval in forested habitats. Properties were not sampled for ticks in 2020 because of the COVID-19 pandemic. (TIF) [file pone.0293820.s005.tif]

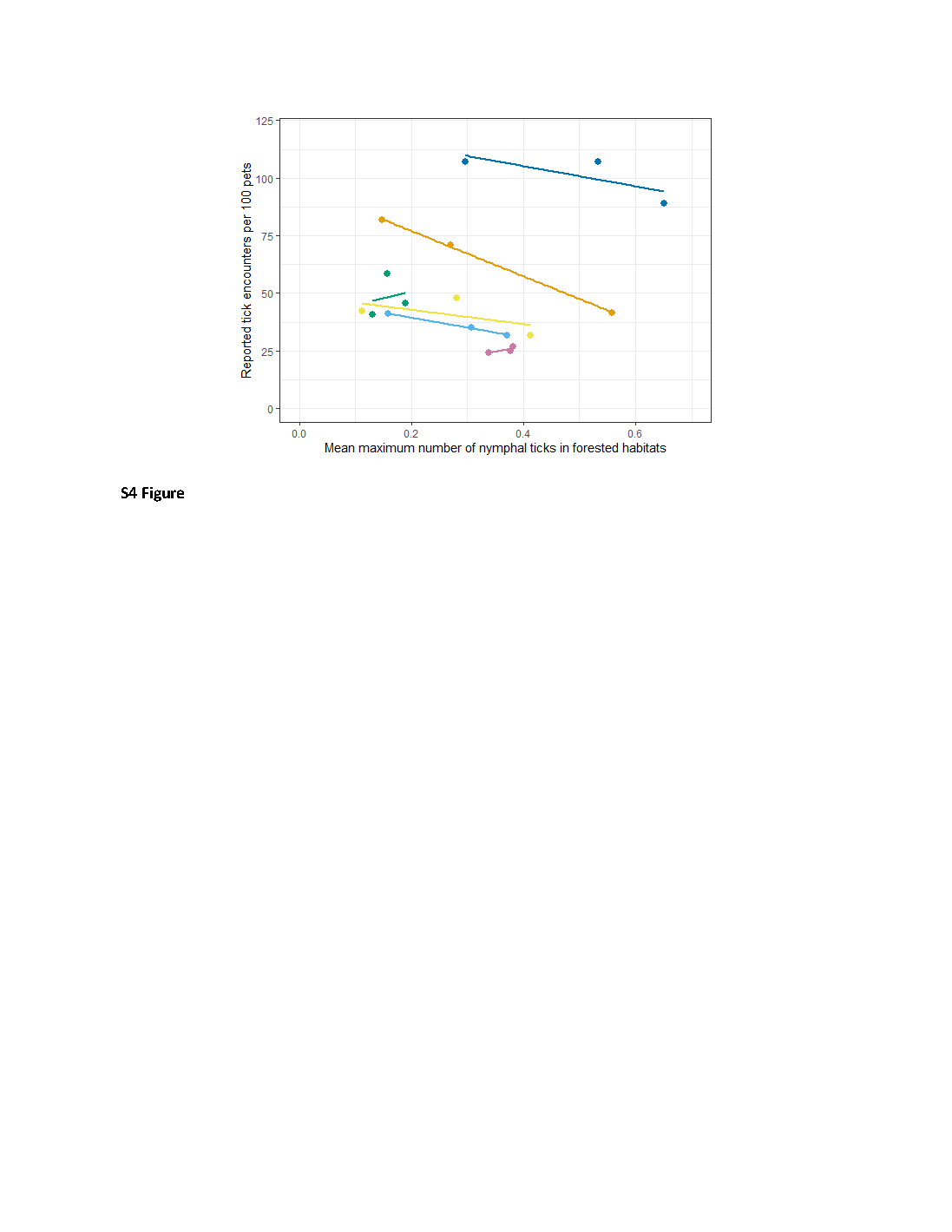

Supplement: S4 Fig — Colors represent the six neighborhoods sampled in three years (2017–2019). Tick encounters were negatively correlated with average tick abundance (χ2 = 14.79, P<<0.01). (TIF) [file pone.0293820.s006.tif]
